# Circulating levels of P-selectin and E-selectin relate to cardiovascular magnetic resonance-derived aortic characteristics in young adults from the general population, a cross-sectional study

**DOI:** 10.1186/s12968-018-0473-8

**Published:** 2018-08-02

**Authors:** Anouk L. M. Eikendal, Michiel L. Bots, Aisha Gohar, Esther Lutgens, Imo E. Hoefer, Hester M. den Ruijter, Tim Leiner

**Affiliations:** 1 Laboratory of Experimental Cardiology, University Medical Center Utrecht, Heidelberglaan 100, 3584 CX Utrecht, The Netherlands; 2 Julius Center for Health Sciences and Primary Care, University Medical Center Utrecht, Utrecht, The Netherlands; 3 Department of Medical Biochemistry, Academic Medical Center Amsterdam, Amsterdam, The Netherlands; 4 Laboratory of Clinical Chemistry and Hematology, University Medical Center Utrecht, Utrecht, The Netherlands; 5 Department of Radiology, University Medical Center Utrecht, Utrecht, The Netherlands

**Keywords:** CMR, MRI, Young adults, Adhesion molecules, Inflammation, Atherosclerosis

## Abstract

**Background:**

Although endothelial cell adhesion molecules (CAMs) are postulated to play a key role in early atherosclerosis, studies on endothelial CAMs are mainly pertained to middle-aged populations and populations with an unfavourable cardiovascular risk burden. Therefore, this study evaluated whether circulating endothelial CAMs are related to cardiovascular magnetic resonance imaging (CMR) derived indicators of arterial wall alterations in a random sample of young adults from the general population.

**Methods:**

This cross-sectional study is part of the general-population-based Atherosclerosis-Monitoring-and-Biomarker-measurements-In-The-YOuNg (AMBITYON) cohort study. In 131 adults (age: 25–35 years), demography, anthropometry and a lipid spectrum was acquired. Thoracic aortic wall area, wall thickness and pulse wave velocity (PWV) were measured using a 3 T CMR-system. From stored blood samples, four CAMs (E-selectin, P-selectin, vascular CAM-1 and intercellular CAM-1) were measured using dedicated methods. Linear mixed-effects regression analysis was used to evaluate the relation of these CAMs with the selected aortic characteristics.

**Results:**

Of the studied endothelial CAMs, P-selectin related to natural logarithm transformed aortic wall thickness (β = 0.18 mm/(μg/ml), [95% confidence interval: 0.04, 0.31], *p* = 0.01) whereas E-selectin related to natural logarithm transformed aortic PWV (β = 3.01 (m/s)/(μg/ml), [95% confidence interval: 0.08, 5.95], *p* = 0.04). Of note, VCAM-1 and ICAM-1 did not relate to the selected aortic characteristics.

**Conclusions:**

In young adults from the general population, circulating P-selectin and E-selectin levels appear positively related to CMR-derived aortic wall thickness and PWV, possibly pointing towards atherogenic inflammatory arterial wall alterations inflicted by these CAMs already in young adulthood.

**Trial registration:**

Netherlands National Trial Register (NTR): NTR4742, Registered 18 August 2014, retrospectively registered.

**Electronic supplementary material:**

The online version of this article (10.1186/s12968-018-0473-8) contains supplementary material, which is available to authorized users.

## Subject Codes

Clinical Studies, Vascular Biology, Magnetic Resonance Imaging (MRI), Atherosclerosis

## Background

Atherosclerosis is a chronic, inflammatory disease that develops from early childhood onwards and progresses silently for decades before evolving into symptomatic cardiovascular disease (CVD) [1]. The initiation and progression of atherosclerosis depends on profound dynamic modifications in arterial biology [2]. A crucial multi-step biological mechanism that is considered a fundamental early promotor of atherosclerosis is an enhanced homing and adherence of leukocytes to the vascular endothelium and their ensuing migration through the arterial wall into its intima where they instigate the migration of smooth muscle cells (SMCs) and the evolution of foam cells and lipid deposits [3].

The multi-step mechanism is induced by atherogenic stimuli (i.e. lipoproteins, inflammatory cytokines) and mediated by various endothelial cell adhesion molecules (CAMs) that are expressed on the surface of endothelial cells [3–5]. Among identified endothelial CAMs, the biological properties and expression of circulating levels of E-selectin, P-selectin, intercellular adhesion molecule (ICAM-1) and vascular cell adhesion molecule (VCAM-1) are well described but remain to be fully studied in various populations [4]. To date, research on endothelial CAMs has mainly been performed in middle to older aged populations and populations with an increased CVD risk burden. Yet, given the early life origins of atherosclerosis, their role in younger, asymptomatic populations warrants exploration.

As in young adults clinical CVD endpoints rarely occur, proxies for detecting subclinical atherosclerosis are required. A high-end modality that may serve this purpose is cardiovascular magnetic resonance imaging (CMR) [6]. In addition to the completely non-invasive obtainment of indices, CMR has a large anatomical coverage and superior soft tissue contrast as well as the ability to combine arterial morphology and function in a single examination and depict tissues deep inside the body. As such, CMR allows for accurate evaluation of the various stages of aortic atherosclerosis [6]. Indeed, various CMR-derived indices such as aortic wall thickness and pulse wave velocity (PWV), have shown to reliably reflect atherosclerosis burden in asymptomatic populations [6]. In young populations, specifically the aorta is an important artery given that it is prone to early atherosclerotic alterations at a young age [1].

In view of the above, the aim of the current study was to explore the relation between circulating endothelial CAMs and CMR-derived indicators of arterial wall alterations in a community-based random sample of young adults in order to improve our understanding of the pathophysiology underlying the relatively early stages of atherosclerosis.

## Methods

### Study design and population

This cross-sectional study is part of the Atherosclerosis-Monitoring-and-Biomarker-measurements-In-The-YOuNg (AMBITYON) cohort study that was initiated in 2014 (Netherlands National Trial Register number: 4742). The rationale as well as a detailed description of the AMBITYON study are described elsewhere [7–9]. In short, the AMBITYON study comprises 131 participants who were randomly selected from Leidsche Rijn, a borough in the city of Utrecht, The Netherlands. To participate in the AMBITYON study, individuals had to be between 25 and 35 years, without history of symptomatic CVD or use of CVD protective medication, cardiac arrhythmias and absolute or relative contra-indications to CMR. The Institutional Review Board (IRB) of the University Medical Center Utrecht approved the AMBITYON study (IRB: 13/397). Before enrollment, written informed consent was obtained from all participants.

### Measurements

#### Demography and anthropometry

As described elsewhere in detail, demographic and anthropometric information was collected [9]. Demographic information was collected via a standardized electronic questionnaire that included queries on the presence of CVD risk factors such as age, sex, smoking status and diabetes status [9]. Furthermore, in each participant, height, weight, body mass index (BMI), mean systolic (SBP) and diastolic blood pressure (DBP) as well as waist and hip circumference were assessed [9]. Of the 131 enrolled participants, the median age was 31.8 years ([25th percentile, 75th percentile (Q1, Q3): 28.9 years, 33.8 years [interquartile range (IQR): 4.9 years]], [min: 25.0 years, max: 35.8 years, [range: 10.8 years]]), 63 (48.1%) were males, 1 (0.8%) participant had diabetes (no use of medication for diabetes) and 28 (21.4%), 23 (17.6%) and 80 (61.0%) participants were current, former and never smokers, respectively. Furthermore, median BMI was 23.2 kg/m^2^ ([Q1, Q3: 21.6 kg/m^2^, 25.0 kg/m^2^ [IQR: 3.4 kg/m^2^]], [min: 17.9 kg/m^2^, max: 33.6 kg/m^2^ [range: 15.7 kg/m^2^]]) and mean SPB was 128 mmHg (standard deviation (SD): 12.0). These as well as the other aforementioned demographic and anthropometric characteristics are listed in Additional file 1: Appendix 1.

#### General CMR protocol

An in depth description of the CMR protocol as well as the assessment of the studied aortic parameters, including reference values, has recently been published elsewhere [7, 8]. In short, in each participant a CMR examination of the thoracic aorta was carried out. Imaging was performed on a 3.0 T multi-transmit clinical CMR system (Achieva, Software Release 5.1.7.2, Philips Healthcare, Best, the Netherlands) using a 32-channel phased-array cardiac receive coil. Imaging was performed with the participant placed in a supine position. Before the CMR examination, each participant was trained in end-expiratory breath holding. Furthermore, the CMR examination was carried out within the standard specific absorption rate (SAR) limits.

#### Assessment of aortic wall geometry

Image assessment and analysis was carried out according to a standardized protocol of which a detailed description can be found in previous publications [7, 8]. In short, geometry of the descending thoracic aortic wall was assessed by acquiring sagittal images of the descending thoracic aorta from the top of the aortic arch to the most distal boundary of the cardiac coil (spanning ~ 35 cm of descending thoracic aorta) using an isotropic, 3-dimensional, T1-weighted, black-blood, turbo-spin-echo, non-contrast-enhanced sequence with variable flip angles (3D-T1-BB-VISTA) with Spectral Attenuated Inversion Recovery (SPAIR) fat suppression and a sensitivity encoding (SENSE) parallel imaging algorithm (for faster image acquisition) during free breathing and without electrocardiogram (ECG) gating [7, 8]. Imaging parameters of the 3D-T1-BB-VISTA sequence are listed in Additional file 1: Appendix 2.

**Fig. 1:**
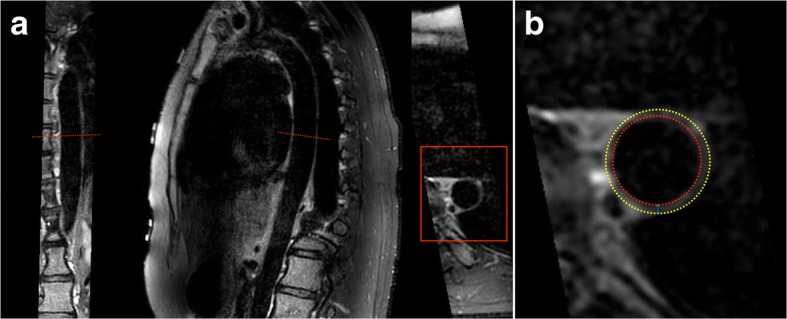
Example of measurement and quantification of aortic wall geometry. Coronal reconstruction (**a**, left), sagittal double oblique source image (**a**, middle) and transverse double oblique reconstruction (**a**, right) of the thoracic aortic vessel wall obtained with the 3D-T1-BB-VISTA acquisition in a 32 year female. In panel **b** aortic wall thickness measurements are illustrated. Vessel wall thickness is calculated by the area of the wall between the red inner red and yellow outer vessel wall contours. Wall thickness is calculated by the distance between the contours (blue arrow). The software VesselMass automatically subdivides the aortic wall into 4 segments. In each segment, 25 aortic wall geometry measurements are performed

Subsequently, descending thoracic aortic wall geometry was quantified using a customized software program specifically developed for measuring geometric dimensions of arterial walls (Vessel Mass, release 5.1, Laboratory for Clinical and Experimental Image processing (LKEB), The Netherlands) [10]. The used quantification methods have previously shown to have an excellent reproducibility for aortic wall geometry assessment (intraclass correlation coefficient (ICC): 0.76–0.99) [7]. An in depth description of image analysis can be found elsewhere [7, 8]. In short, aortic wall geometry was assessed using the images between the origin of the descending thoracic aorta and the origin of the celiac trunk. A sample of the first 50 participants showed that, on average, in each participant ~ 22 cm (cm) of aorta was analysed (mean: 21.9 cm, [SD: 1.8], median: 22.0 cm [Q1: 21.0, Q3: 23.0]). Per cm of aorta, 1 image was assessed for aortic wall geometry via manual tracing of the aortic wall boundaries by an experienced researcher (AE, 3 years experience in aortic wall geometry assessment). On average, aortic wall geometry was assessed in 22 images per participant. Based on the traced contours, the software Vessel Mass automatically computed the mean aortic wall area and thickness in each participant according to a previously described method by summing the mean aortic wall area (cm^2^) and thickness (mm) for all slices in that participant and divide it by the number of quantified slices in that participant [7, 10]. Fig. 1 provides examples of descending thoracic aorta CMR-images as well as a schematic representation of aortic wall geometry assessment.

#### Assessment of thoracic aortic pulse wave velocity

To evaluate thoracic aortic stiffness, PWV was evaluated over the entire thoracic aorta as described in depth elsewhere [8, 9]. In short, to enable measurement of the length of the thoracic aorta, the full course of the aorta was imaged in the sagittal orientation using an ECG-gated double oblique single-slice balanced turbo field gradient-echo sequence with end-expiratory breath holding. Successively, based on this image, the through-plane velocity in the ascending, the proximal descending (acquisition 1) as well as the distal descending thoracic aorta (acquisition 2) was assessed by planning two phase contrast acquisitions with velocity-encoding (VE) perpendicular to the center lumen line of the aorta. The through-plane flow velocities of both acquisitions were collected using a VE, one-directional, through-plane, non-segmented, gradient turbo field echo pulse sequence (VE: 1.5 m/s) in the transversal orientation during free breathing and with retrospective ECG-gating. Per cardiac cycle, 50 heart phases were reconstructed, thereby generating 50 images (interpolation: 50%, temporal resolution (TR): 10–20 ms, subject to heart rate, turbo field echo shot duration: 38.6 ms, actual TR: 76.2 ms) [8, 9]. Imaging parameters of the VE sequences are listed in Additional file 1: Appendix 2.

**Fig. 2:**
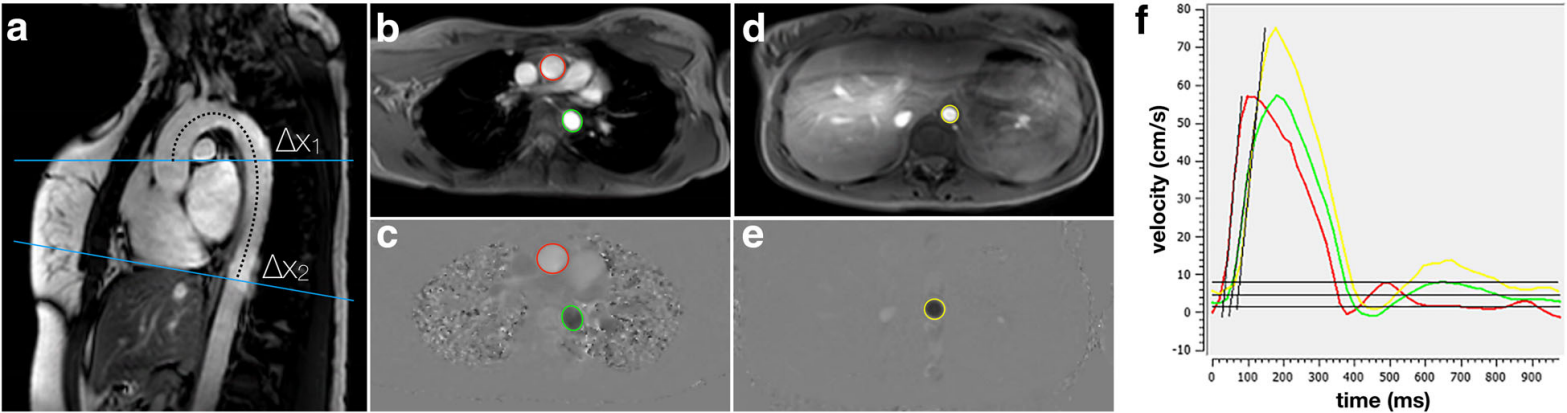
Examples of double-oblique and through-plane velocity-encoded images of the thoracic aorta and of pulse wave velocity (PWV) quantification. Sagittal double oblique acquisition (panel **a**) serves as the input for planning velocity-encoded acquisitions at the level of the ascending (top blue line in **a** and red contours in **b** and **c**) and descending aorta (green contours in **b** and **c**). A more distant cross section at the level of the diaphragm is also acquired (bottom blue line in **a**, and yellow contours in **d** and **e**). PWV = Δx/Δt, where Δx is the distance between the ascending aorta and the proximal descending aorta (Δx1) or the aorta at the level of the diaphragm (Δx2), and Δt is the time difference between the two velocity-time curves as plotted in (**f**)”

Subsequently, PWV was computed as x/t (m/s) with x being the aortic length between the ascending, proximal descending and distal descending thoracic aorta as measured along the centre lumen line and t being the time duration between the arrival of the systolic pulse wave at these locations. PWV was quantified utilizing validated, semi-automated tailored software program for measurement of PWV (MASS version 5.1, LKEB, Leiden, The Netherlands) and conforming to a standardized, validated procedure that has an excellent inter- scan, inter-rater and intra-rater reproducibility (ICC: 0.87–0.92) [8, 9, 11]. An experienced researcher (AE, 3 years experience in PWV assessment) measured the distance between the flow measurement planes in the ascending to proximal descending, proximal descending to distal descending and total thoracic aorta within the double oblique image by manually tracing the aorta along its centreline between each measurement site. Subsequently, using a semi-automatic flow analysis tool in MASS, the outer contours of the ascending and proximal descending (VE acquisition 1) as well as the distal descending (VE acquisition 2) thoracic aorta were traced in all 50 images of the two VE acquisitions to generate two aortic velocity maps. From these aortic velocity maps, a second customized software program (PwvAppStatic, LKEB, Leiden, The Netherlands) generated velocity-time graphs that were used to calculate the time delay between the arrival of the systolic pulse wave at the three measurement sites. By combining the time delay and aortic lengths, the software PwvAppStatic computed the absolute PWV value (m/s) in each of the three measurement sites via linear regression modelling. Figure 2 provides an example of the double oblique and through-plane VE images of the thoracic aorta as well as an illustration of PWV quantification.

#### Laboratory assessments

Before the CMR examination, a venous blood sample was collected in each participant. This sample was obtained in 3 tubes: an EDTA tube (3 ml), a sodium-heparin tube (2 ml) and a serum tube (5 ml). The serum sample was centrifuged one hour after collection of the sample for 10 min at 1850×g and a temperature of 21 °C. Subsequently, the serum fraction was isolated and stored at − 80 °C. The serum samples remained stored until they were thawed for further analysis [9].

##### Lipid and glucose levels

The Laboratory of Clinical Chemistry of the UMC Utrecht instantly processed the EDTA and sodium-heparin sample and measured glucose, triglycerides, total cholesterol and high-density lipoprotein (HDL)-cholesterol levels using a routine clinical chemistry analyser (AU5811, Beckman Coulter, Brea, California, USA). The Friedewald formula was used to calculate the low-density lipoprotein (LDL)-cholesterol level [12] The distribution of the studied lipid and glucose levels is listed in Additional file 1: Appendix 1.

##### Endothelial cell adhesion molecules

The circulating levels of P-selectin, E-selectin, VCAM-1 and ICAM-1 were measured at the Multiplex Core Facility of the UMC Utrecht with an in-house developed, validated and highly reproducible multiplex immunoassay based on xMAP technology (Luminex, Austin, Texas, USA) that is set to analyse batches of up to 80 samples per multi-well micro plate. A detailed description of this procedure has been published elsewhere [13]. Briefly, after the serum samples in all participants were collected, the samples were thawed to room temperature for further analysis. Then, to pre-absorb a-specific heterophilic immunoglobulins in all samples, heteroblock (Omega Biologicals, Bozeman, Montana, USA) was used. To carry out acquisition, a combination of Biorad FlexMAP3D (Biorad Laboratories, Hercules, California, USA) and xPONENT software version 4.2 (Luminex) was used. The acquired information was analysed with 5-parametric curve fitting using Bio-Plex Manager software version 6.1.1 (Biorad). Intra- and inter-assay coefficients of variation for P-selectin, E-selectin, ICAM-1 and VCAM-1 were 2.9 (±2.1) and 7.1 (±1.9), 3.8 (±2.0) and 11.5 (±2.4), 5.1 (±3.6) and 9.2 (±2.5), 3.1 (±1.7) and 13.1 (±4.9), respectively.

#### Missing data

In 124/131 (94.7%) participants, aorta imaging was successful. In 118 of these 124 (95.2%) participants, image quality was sufficient for PWV quantification. Unfortunately, in 7, 4, 4 and 6 participants respectively, measurement of P-selectin, E-selectin, ICAM-1 and VCAM-1 failed due to insufficient material in the obtained samples whereas in 6 and 5 participants in whom aorta imaging was successful and PWV was quantified respectively, these missing values were overlapping. Therefore, complete case analysis was performed in 118/131 (90.1%) participants for aortic wall geometry and in 113/131 (85.5%) participants for aortic PWV.

### Data analysis

Linear mixed-effects regression analysis was used to study the relation between the circulating endothelial biomarkers and mean aortic wall area, wall thickness and PWV. For this analysis, we constructed 2 models; a crude model (Model 1) and a multivariable model (Model 2), in which we adjusted Model 1 for the a priori selected possible confounders age (years), sex (reference: male), smoking (current/former vs. never), BMI (kg/m^2^), DBP (mm Hg) and total and HDL-cholesterol level (both in mmol/L). We did not adjust our models for SBP and LDL-cholesterol since SBP and DBP and total and LDL-cholesterol were highly correlated (Spearman’s *r* = 0.75 and 0.89, respectively). Additionally, we performed an analysis in which we compared the current/former smoking population to the never smoking population using the same method as mentioned above. In all analyses, we adjusted for batch as a random effects term in all models to correct for heterogeneity between batches. All other covariates were used as fixed effects. For the analyses, the aortic characteristics were log transformed to normalize skewed distributions and meet the mandatory criteria for linear regression models. Conclusions were based on linear mixed-effects regression coefficients (beta’s (β)) with 95% confidence intervals (CI), conforming to p_2 sided_ < 0.05; which was considered statistically significant. In addition, for the multivariable relation between the circulating endothelial biomarkers and aortic characteristics, 8 variables were entered in each model (endothelial CAM + adjustment for age, sex, smoking, BMI, DBP, total and HDL-cholesterol). As such, we calculated that we respectively required a minimum sample size of *n* = 76 and *n* = 108 to provide reliable estimates using a power of 80%, an alpha level of 5% and a medium effect size of 0.15. Sample size calculations were performed using Daniel Soper’s Sample Size Calculator (http://www.danielsoper.com/statcalc3/calc.aspx?id=1). All other data analyses were performed with SPSS version 21.0 (International Business Machines, Armonk, New York, USA).

## Results

### General

In the 124 subjects in whom aortic wall geometry was successfully assessed, mean aortic wall area and median aortic wall thickness were 1.0 cm^2^ (±0.1) and 1.5 mm (1.4, 1.7), respectively. In the 118 subjects in whom aortic PWV was successfully assessed, median aortic PWV was 4.4 m/s (4.1, 4.8). The distributions of the studied circulating endothelial biomarkers are listed in Additional file 1: Appendix 1.

### Relation between circulating endothelial biomarkers and aortic characteristics

The crude model, Model 1, showed that P-selectin significantly and positively related to both aortic wall area (β = 0.21 cm^2^ per 1 μg/ml increase [95% CI: 0.04, 0.38], *p* = 0.02) and log-aortic wall thickness (β = 0.19 mm per 1 μg/ml increase [95% CI: 0.06, 0.32], *p* = 0.005). Additionally, Model 1 demonstrated a significant, positive relation between E-selectin and log-aortic PWV (β = 3.83 m/s per 1 μg/ml increase [95% CI: 1.14, 6.53], *p* = 0.006) (Table 1). After full adjustment (Model 2), P-selectin did not relate significantly to aortic wall area anymore but was still significantly and positively related to log-aortic wall thickness (β = 0.18 mm per 1 μg/ml increase [95% CI: 0.04, 0.31], *p* = 0.01). Model 2 also showed that the relation between E-selectin and log-aortic PWV remained significant and positive after full adjustment (β = 3.01 m/s per 1 μg/ml increase [95% CI: 0.08, 5.95], *p* = 0.04). Of note, we did not observe any relation of VCAM-1 and ICAM-1 with the aortic characteristics (Table 1).

**Table 1:** Relation between circulating endothelial cell adhesion molecules (CAMs) and aortic characteristics

	Aortic wall area (cm^2^)^b^ (*n* = 124)	*p* value	Aortic wall thickness (mm)^b,d^ (*n* = 124)	*p* value	Aortic PWV (m/s)^b,d^ (*n* = 118)	*p* value
P-selectin (μg/ml)^c^						
Model 1	0.21 (0.04, 0.38)	0.02§	0.19 (0.06, 0.32)	0.005§	−0.09 (− 0.32, 0.13)	0.41
Model 2	0.14 (−0.02, 0.30)	0.08	0.18 (0.04, 0.31)	0.01§	−0.17 (− 0.39, 0.54)	0.14
E-selectin (μg/ml)^c^						
Model 1	0.01 (−2.26, 2.28)	0.99	0.26 (−1.46, 1.99)	0.77	3.83 (1.14, 6.53)	0.006§
Model 2	−1.72 (−3.84, 0.39)	0.11	−0.45 (−2.28, 1.39)	0.63	3.01 (0.08, 5.95)	0.04§
ICAM − 1 (μg/ml)^a,c^						
Model 1	0.15 (−0.02, 0.32)	0.08	0.11 (−0.02, 0.24)	0.08	0.09 (−0.11, 0.30)	0.36
Model 2	0.15 (−0.05, 0.26)	0.18	0.11 (−0.03, 0.24)	0.12	0.02 (−0.24, 0.27)	0.88
VCAM-1 (μg/ml)^a,c^						
Model 1	0.01 (−0.01, 0.03)	0.36	-1.59 × 10^− 3^ (− 0.02, 0.01)	0.85	− 0.01 (− 0.03, 0.01)	0.41
Model 2	−2.03 × 10^−3^ (− 0.02, 0.02)	0.84	−2.73 × 10^− 3^ (− 0.02, 0.01)	0.75	−0.01 (− 0.03, 0.01)	0.53

^a^*PWV* pulse wave velocity, *ICAM-1* intercellular adhesion molecule, *VCAM-1* vascular cell adhesion molecule

^b^Values are linear mixed-effects regression coefficients (beta’s, (β)) with 95% confidence intervals

^c^Model 1: crude model, Model 2: adjusted for age, sex, BMI, smoking, DBP, HDL-cholesterol and total cholesterol

§ *p* < 0.05

^d^Natural logarithmic transformation was performed

Interestingly, when the population was stratified for smoking, the crude (Model 1) and multivariable (Model 2) model showed that the aforementioned significant associations of P-selectin with log-aortic wall thickness and E-selectin with log-aortic PWV only remained significant in the current/former smoking population (Additional file 1: Appendix 3).

Finally, the addition of multiplicative interaction terms showed that there was no effect modification by sex (*p* > 0.10 for all comparisons). Therefore, analyses and results were not stratified for sex.

## Discussion

This study expands current knowledge on the relation of endothelial CAMs with arterial wall alterations in young adults from the general population by showing that already in a young population an increase in circulating P-selectin and E-selectin relate to an increase in CMR-derived aortic characteristics, possibly with a important role for smoking. Our results suggest that upregulation of P-selectin, and to a lesser extent, E-selectin may mirror atherogenic inflammatory alterations in the vascular bed. This study may contribute to an improved understanding of the biology and determinants of early atherosclerosis and thus may possibly aid in developing effective interventions when atherosclerosis is still, at least partially, reversible.

Soluble forms of CAMs can be detected in the circulation due to their release from the endothelium via shedding or proteolytic cleavage [14, 15]. Although their biological role is not yet fully identified, soluble CAMs appear to reliably mirror increased expression of membrane-bound CAMs and reflect the inflammatory component of atherosclerosis [14, 15]. Although studies report discordant results, CAMs seem involved in CVD pathophysiology. Their levels rise in relation to various CVD risk factors. Additionally, they have been related to morphological and functional measures of atherosclerosis as well as to an unfavourable CVD prognosis in various populations [4]. For example, positive relations between all four CAMs and age, BMI, blood pressure and lipid levels have been reported [16]. Additionally, for P-selectin, E-selectin and ICAM-1, higher levels have been observed in smokers as compared to non-smokers. [16–18]. Moreover, studies have reported positive relations of P-selectin and E-selectin with carotid intima-media thickness (CIMT), arterial stiffness, plaque burden and presence of clinically overt CVD in various low and high-risk populations [14]. For VCAM-1 and ICAM-1 relations have been observed with CIMT, plaques and clinically overt CVD [14]. Yet, others failed to observe such relations [19].

In this study, both the crude and multivariable model showed that P-selectin and E-selectin positively related to aortic wall thickness and aortic stiffness, respectively. This indicates that the a priori selected confounding variables, known to be risk factors for atherosclerosis and related to the CAMs, exerted little effect on the observed associations, assuming these variables are not in the causal pathway between CAMs and aortic characteristics. However, the relation of P-selectin with aortic wall area was significant in the crude model but lost its significance in the multivariable model, with a substantial change in regression coefficient (> 30%), implying that confounding biased the relation of P-selectin with aortic wall area by increasing the effect of the association.

Interestingly, when current/former smokers were compared to never smokers, the significant associations only remained significant in the current/former smoking population. Although these results have to be interpreted with care given the relatively small study population, they suggest effect modification of the association between CAMs and aortic characteristics by smoking. In addition to being an established risk factor for atherosclerosis, smoking may prompt vascular inflammation as it increases endothelial adhesion and yields pathological production of endothelial vasoactive substances, including endothelial generation and expression of adhesion molecules [20–23]. Indeed, studies have documented a larger variation in CAMs, including P-selectin and E-selectin, in smokers as compared to non-smokers [17, 20–23]. This increase in CAMs may facilitate the recruitment of leukocytes and other inflammatory mediators to locations of arterial damage and as such, may inflict atherogenic vascular inflammation [18, 22, 23].

In this study, VCAM-1 and ICAM-1 did not relate to the selected aortic characteristics. This may be due to the varying expression and release of CAMs, depending on the sources and arterial beds they are derived from as well as to intrinsic differences in the biological function of endothelial CAMs, the (CVD risk) factors affecting their levels and the shedding process. For example, whereas the selectins are mostly involved in recruitment and rolling of leukocytes, VCAM-1 and ICAM-1 mainly promote binding and transmigration of leukocytes into the arterial intima, where they exert their noxious effects [18]. Furthermore, VCAM-1 appears to be specifically upregulated in regions of intimal neovascularization and advanced atherosclerotic lesions, which may clarify the lack of an association with relatively early arterial wall alterations [24]. Moreover, whereas E-selectin is derived solely from endothelial cells, the other CAMs are derived from multiple sources such as platelets (P-selectin), leukocytes (ICAM-1) and cell types of non-vascular origin (VCAM-1), which may explain the strong association of E-selectin with aortic PWV [25]. Additionally, heterogeneity in studied population and used methodology also yield discrepancies in results across studies and may explain why we did not observe significant associations of VCAM and ICAM with the studied aortic characteristics.

Reducing CVD risk factors in the young may possibly reverse the atherogenic process. Prior intervention studies indeed observed that interventions, such as a diet, smoking cessation or antihypertensive medication use, did not only reduce lipid levels, weight and blood pressure, but also induced a decline in levels of the related CAMs [18, 21, 26, 27]. Unfortunately, to date, the involvement of circulating endothelial CAMs in atherosclerosis pertains to be complex, multifaceted and not yet fully understood. CVD risk factors, atherogenic stimuli, inflammatory mediators and the endothelium all appear to be tangled in a vicious circle that induces harmful arterial wall alterations. This study indicates that P-selectin, E-selectin and smoking play a central role already in the early stages of this vicious circle. Nevertheless, the ambiguous results across studies seem to advocate that, in addition to developing a standardized methodology for these type of studies, further unravelling of the mechanisms, biology and determinants of circulating endothelial CAMs is merited before hard conclusions can be drawn. Therefore, we believe that our results warrant further exploration in larger cohorts.

Strengths of this study are our random selection of young adults from the general population, the use of promising, highly innovative imaging based indicators of arterial wall alterations and the measurement of multiple endothelial biomarkers. Moreover, in young individuals, confounding effects of lifestyle on the levels of the endothelial biomarkers are limited as compared to middle-aged to older individuals. However, limitations of this study also merit attention. Although this is one of the largest studies comparing biomarkers with CMR-derived indices of arterial wall alterations, our limited sample size forced us to interpret our results with care. Chance as well as residual confounding may have played a role, yet the latter can never be excluded in cohort studies like this. Also, due to the limited spatial resolution of MRI, arterial wall alterations that may have been present but are yet too subtle to be detected with CMR (i.e. intimal thickening) may have remained undetected. Technical improvements such as calibration of imaging protocols, standardization and automation of quantification methods as well as a refinement in spatial resolution are warranted. Moreover, although recruitment was random, sampling bias may have influenced our results; health-conscious individuals may have been more eager to participate, which may have induced an underestimation of our results. In addition, our study population comprised Caucasian, young adults with a relatively large percentage of current/former smokers. As such, the features of our study population restrict the generalizability of our results to populations that are similar to our study population. Also, the cross-sectional nature of our study prevented us from making inferences on causality of the studied relations. Moreover, we assessed only 4 endothelial biomarkers, yet other unknown molecules and pathways are undeniably involved in the multi-step mechanism.

## Conclusions

In conclusion, circulating P-selectin and E-selectin levels seem related to CMR-derived arterial wall alterations and as such, may be involved in the development of atherogenic inflammatory arterial wall alterations already in young adults from the general population.

## Additional file

Additional file 1: **Appendix 1.** Characteristics of study population (N = 131). **Appendix 2.** MR Imaging parameters of 3D-T1-BB-VISTA and velocity encoded sequences. **Appendix 3.** Relation of circulating endothelial CAMs with aortic characteristics stratified for current/former smoking and never smoking. (DOCX 30 kb)

## Abbreviations

AMBITYON: Atherosclerosis-Monitoring-and-Biomarker-measurements-In-The-YOuNg
BMI: Body mass index
CAMs: Cell adhesion molecules
CI: Confidence interval
CIMIT: Carotid intima-medial thickness
CMR: Cardiovascular magnetic resonance
CVD: Cardiovascular disease
DBP: Diastolic blood pressure
ECG: Electrocardiogram
HDL: High-density lipoprotein
ICAM-1: Intercellular adhesion molecule 1
ICC: Intraclass correlation coefficient
IRB: Institutional Review Board
LDL: Low-density lipoprotein
PWV: Pulse wave velocity
SAR: Specific absorption rate
SBP: Systolic blood pressure
SD: Standard deviation
SENSE: Sensitivity encoding
SMCs: Smooth muscle cells
SPAIR: Spectral attenuated inversion recovery
T: Tesla
VCAM-1: Vascular cell adhesion molecule 1
VE: Velocity-encoding
