# Supplementary material for: Circulating levels of P-selectin and E-selectin relate to cardiovascular magnetic resonance-derived aortic characteristics in young adults from the general population, a cross-sectional study
Source: J Cardiovasc Magn Reson. 2018 Aug 2;20:54. doi: 10.1186/s12968-018-0473-8 (PMC6090925; doi:10.1186/s12968-018-0473-8)
Supplement: Supplementary file 1 — Appendix 1. Characteristics of study population (N = 131). Appendix 2. MR Imaging parameters of 3D-T1-BB-VISTA and velocity encoded sequences. Appendix 3. Relation of circulating endothelial CAMs with aortic characteristics stratified for current/former smoking and never smoking. (DOCX 30 kb) [file 12968_2018_473_MOESM1_ESM.docx]

| **Supplemental Appendix 1.** Characteristics of study population (N = 131) | | |
| --- | --- | --- |
|  | **N** | **Total population** |
| **Demographic characteristics** |  |  |
| Age (years), median (Q1, Q3)* | 131 | 31.8 (28.9, 33.8) |
| Sex (men), *n* (%) | 131 | 63 (48.1) |
| Smoking status, *n* (%)   - current - former - never | 131 | 28 (21.4)  23 (17.6)  80 (61.0) |
| Diabetes mellitus (yes), *n* (%) | 131 | 1 (0.8%) |
| **Anthropometric characteristics** | | |
| Length (cm), mean (±SD)* | 131 | 176.9 (±8.9) |
| Weight (kg), mean (±SD)* | 131 | 73.6 (±11.5) |
| Waist circumference (cm), mean (±SD)* | 131 | 79.6 (±8.7) |
| Hip circumference (cm), mean (±SD)* | 131 | 87.6 (±8.5) |
| BMI (kg/m^2^), median (Q1, Q3)* | 131 | 23.2 (21.6, 25.0) |
| SBP (mm Hg), mean (±SD)* | 131 | 128.0 (±12.0) |
| DBP (mm Hg), mean (±SD)* | 131 | 79.0 (±8.0) |
| **Lipid and glucose levels** | | |
| Total cholesterol level (mmol/L), mean (±SD)* | 129 | 4.6 (±0.8) |
| HDL-cholesterol level (mmol/L), median (Q1, Q3)* | 129 | 1.4 (1.2, 1.7) |
| LDL-cholesterol level (mmol/L), mean (±SD)* | 125 | 2.6 (±0.7) |
| Triglyceride level (mmol/L), median (Q1, Q3)* | 129 | 1.2 (0.9, 1.8) |
| Glucose level (mmol/L), median (Q1, Q3)* | 129 | 5.1 (4.7, 5.5) |
| **Circulating endothelial biomarkers** | | |
| P-selectin (µg/ml), median (Q1, Q3)* | 124 | 0.1 (0.1, 0.2) |
| E-selectin (µg/ml), mean (±SD)* | 127 | 0.2 (±0.01) |
| ICAM-1 (µg/ml), median (Q1, Q3)* | 127 | 0.4 (0.3, 0.6) |
| VCAM-1 (µg/ml), median (Q1, Q3)* | 125 | 2.3 (1.8, 3.3) |

* Q1: 25^th^ percentile, Q3: 75^th^ percentile, SD: standard deviation, BMI: body mass index, SBP: systolic blood pressure, DBP: diastolic blood pressure, HDL: high-density lipoprotein, LDL: low-density lipoprotein, ICAM-1: intercellular adhesion molecule, VCAM-1: vascular cell adhesion molecule

**Supplemental Appendix 2. MR Imaging parameters of 3D-T1-BB-VISTA and velocity encoded sequences**

|  | **3D-T1-BB-VISTA**  *(aortic wall geometry)* | **Velocity encoded sequence**  *(aortic pulse wave velocity)* |
| --- | --- | --- |
| **Field of view (mm)** | 350x302x45 | 320x350 |
| **Acquired spatial resolution (mm)** | 1.20x1.20x1.20 (mm^3^) | 2.50x2.50 (mm^2^) |
| **Reconstructed spatial resolution (mm)** | 0.60x0.60x0.60 | 1.25x1.25 |
| **Echo time (ms)** | 33.00 | 2.90 |
| **Repetition time (ms)** | 1000.00 | 4.80 |
| **Turbo spin echo factor / Turbo field echo factor** | 45.00 | 9.00 |
| **Flip angle (°)** | 90 (refocusing α_min_=20°, α_max_=112°) | 10 |
| **Number of signal averaging (n)** | 2 | 1 |
| **Number of slices (n)** | 75 | 1 (50 heart phases) |
| **Slice thickness (mm)** | 1.2 | 8 |
| **Slice gap (mm)** | None | None |
| **Acquisition duration (minutes : seconds)** | 7:38 | depending on heart rate |
| **ECG triggering** | no | yes (retrospective) |
| **Breath holding** | no | no |

| **Supplemental Appendix 3.** Relation of circulating endothelial CAMs with aortic characteristics stratified for current/former smoking and never smoking | | | | | | |
| --- | --- | --- | --- | --- | --- | --- |
|  | **Aortic wall area (cm^2^)**† (*n* = 124) | | **Aortic wall thickness (mm)**†\|\| (*n* = 124) | | **Aortic PWV (m/s)**†\|\| (*n* = 118) | |
|  | *current / former smoking*  *(n = 50)* | *never smoking*  *(n = 74 )* | *current / former smoking*  *(n = 50)* | *never smoking*  *(n = 74)* | *current / former smoking*  *(n = 46)* | *never smoking*  *(n = 72)* |
| **P-selectin (µg/ml)**‡ | | | | | | |
| Model 1 | 0.29 (-0.01, 0.59)  P value: 0.06 | 0.14 (-0.06, 0.34)  P value: 0.15 | 0.33 (0.11, 0.55)  P value: 0.004§ | 0.07 (-0.09, 0.23)  P value: 0.36 | -0.15 (-0.60, 0.30)  P value: 0.50 | -0.03 (-0.27, 0.20)  P value: 0.77 |
| Model 2 | 0.23 (-0.05, 0.51)  P value: 0.11 | 0.09 (-0.11, 0.29)  P value: 0.39 | 0.29 (0.06, 0.52)  P value: 0.016§ | 0.07 (-0.10, 0.24)  P value: 0.42 | -0.23 (-0.70, 0.23)  P value: 0.31 | -0.07 (-0.30, 0.16)  P value: 0.55 |
| **E-selectin (µg/ml)**‡ | | | | | | |
| Model 1 | -1.18 (-5.75, 3.38)  P value: 0.60 | 1.49 (-1.02, 4.02)  P value: 0.24 | -1.15 (-4.18, 2.42)  P value: 0.52 | 1.31 (-0.65, 3.28)  P value: 0.19 | 6.88 (1.39, 12.37)  P value: 0.015§ | 1.95 (-0.87, 4.77)  P value: 0.17 |
| Model 2 | -2.84 (-6.86, 1.16)  P value: 0.16 | 0.16 (-2.75, 3.06)  P value: 0.92 | -2.05 (-5.86, 1.49)  P value: 0.25 | 1.28 (-1.18, 3.74)  P value: 0.30 | 6.89 (1.39, 12.39)  P value: 0.015§ | -0.03 (-3.38, 3.32)  P value 0.99 |
| **ICAM-1 (µg/ml)***‡ | | | | | | |
| Model 1 | 0.10 (-0.21, 0.41)  P value: 0.49 | 0.12 (-0.07, 0.31)  P value: 0.20 | 0.12 (-0.11, 0.35)  P value: 0.29 | 0.09 (-0.07, 0.24)  P value: 0.27 | 0.07 (-0.39, 0.52)  P value: 0.72 | 0.02 (-0.43, 0.47)  P value: 0.84 |
| Model 2 | 0.13 (-0.13, 0.40)  P value: 0.33 | 0.09 (-0.10, 0.28)  P value: 0.34 | 0.13 (-0.10, 0.36)  P value: 0.27 | 0.09 (-0.07, 0.26)  P value: 0.27 | -0.01 (-0.33, 0.32)  P value: 0.96 | -0.03 (-0.19, 0.14)  P value: 0.74 |
| **VCAM-1 (µg/ml)***‡ | | | | | | |
| Model 1 | 0.01 (-0.04, 0.05)  P value: 0.72 | 2.74x10^-3^ (-0.02, 0.03)  P value: 0.82 | -6.29x10^-5^ (-0.03, 0.03)  P value: 0.99 | -0.01 (-0.03, 0.01)  P value: 0.50 | -6.55x10^-4^ (-0.35, 0.35)  P value: 0.98 | -0.01 (-0.04, 0.01)  P value: 0.19 |
| Model 2 | -4.59x10^-3^ (-0.04, 0.03)  P value: 0.78 | -0.01 (-0.03, 0.02)  P value: 0.51 | 8.61x10^-4^ (-0.03, 0.03)  P value: 0.95 | -0.01 (-0.03, 0.01)  P value: 0.48 | 0.01 (-0.08, 0.10)  P value: 0.64 | -0.01 (-0.04, 0.01)  P value: 0.20 |

* PWV: pulse wave velocity, ICAM-1: intercellular adhesion molecule, VCAM-1: vascular cell adhesion molecule

† Values are linear mixed-effects regression coefficients (beta’s, (β)) with 95% confidence intervals and p values

‡ Model 1: crude model, Model 2: adjusted for age, sex, BMI, DBP, HDL-cholesterol and total cholesterol

§ p<0.05

|| Natural logarithmic transformation was performed
